# NeuroCOVID Burden and Functional Impairment in Adults with Long COVID: A Secondary Analysis of an Italian Cohort

**DOI:** 10.3390/jcm15156027

**Published:** 2026-08-03

**Authors:** Angelo Cianciulli, Emanuela Santoro, Roberta Manente, Mariachiara Figura, Antonietta Pacifico, Giovanni Boccia

**Affiliations:** 1 Department of Medicine, Surgery and Dentistry “Scuola Medica Salernitana”, University of Salerno, 84081 Salerno, Italy; ancianciulli@unisa.it (A.C.); apacifico@unisa.it (A.P.); gboccia@unisa.it (G.B.); 2 Division of Laboratory Medicine, Civil Hospital ‘Maria SS. Addolorata’, Local Health Authority of Salerno, 84025 Eboli, Italy; manente392@gmail.com; 3 Department of Maternal and Child Health Promotion, Internal Medicine & Specialties of Excellence “G. D’Alessandro” (PROMISE), University of Palermo, 90133 Palermo, Italy; mariachiara.figura@unipa.it; 4 Integrated Care Department of Health Hygiene and Evaluative Medicine, San Giovanni di Dio e Ruggi D’Aragona University Hospital, 84131 Salerno, Italy; 5 Hospital and Epidemiological Hygiene Unit, San Giovanni di Dio and Ruggi D’Aragona University Hospital, 18 Hospital, 84131 Salerno, Italy

**Keywords:** Long COVID, Post-COVID-19 Condition, NeuroCOVID, cognitive dysfunction, functional impairment, rehabilitation, public health, SARS-CoV-2

## Abstract

**Background/Objectives**: Long COVID is frequently characterized by neurological, cognitive, neuropsychiatric, and autonomic symptoms (NeuroCOVID). However, their impact on daily functioning remains insufficiently investigated. This study evaluated the association between NeuroCOVID burden and functional impairment among Italian Long COVID survivors. **Methods**: A secondary cross-sectional analysis was conducted on 250 adults using a validated Italian Long COVID questionnaire. A NeuroCOVID Burden Score (10 symptom domains) and a Functional Impairment Score (daily activities) were constructed and analyzed using correlation and multivariable linear regression. **Results**: The sample (mean age 33.2 years; 63.6% female) demonstrated high internal consistency for both scores. A strong positive correlation was found between NeuroCOVID burden and functional impairment (r = 0.703; *p* < 0.001). **Conclusions**: Persistent NeuroCOVID burden was strongly associated with greater limitations in daily activities. In multivariable regression, NeuroCOVID Burden Score remained independently associated with functional impairment (B = 0.671; 95% CI 0.566–0.776; *p* < 0.001), together with acute COVID-19 severity (B = 0.156; 95% CI 0.055–0.257; *p* = 0.003). The final model explained 52.5% of the variance in functional impairment (R^2^ = 0.525).

## 1. Introduction

Post-COVID-19 condition (PCC), commonly known as Long COVID, has emerged as one of the most important long-term public health consequences of the SARS-CoV-2 pandemic. In 2021, the World Health Organization (WHO) defined PCC as a condition occurring in individuals with a history of probable or confirmed SARS-CoV-2 infection, usually three months from the onset of COVID-19, characterized by symptoms lasting at least two months and not explained by an alternative diagnosis. Symptoms may be persistent, relapsing, or fluctuating over time and can substantially affect daily functioning and quality of life [1].

Growing evidence indicates that Long COVID is a complex multisystem disorder whose neurological manifestations have become an increasing focus of clinical and public health research [2,3]. Recent estimates suggest that persistent symptoms substantially affect daily functioning, work productivity, social participation, and health-related quality of life [2,3,4,5]. The impact of Long COVID extends beyond healthcare utilization and includes reduced work productivity, impaired social participation, functional limitations, and deterioration in health-related quality of life [4,5].

NeuroCOVID encompasses persistent neurological, cognitive, neuropsychological, neurosensory, and autonomic manifestations that may persist for months after acute SARS-CoV-2 infection [6,7]. Common manifestations include headache, cognitive dysfunction (“brain fog”), concentration difficulties, memory impairment, sleep disturbances, anxiety, depression, dysautonomia, olfactory dysfunction, and other neurosensory alterations [6,7,8,9]. These symptoms are frequently reported even among non-hospitalized individuals and patients who experienced mild acute disease, suggesting that long-term self-reported neurological and neuropsychological manifestations are not restricted to severe COVID-19 cases [8,10].

Recent studies have demonstrated that cognitive impairment represents one of the most disabling dimensions of Long COVID [11,12]. Persistent deficits involving attention, executive functioning, processing speed, and memory have been documented months after the acute infection [11,12,13]. Neuroimaging studies have identified structural and microstructural alterations affecting several brain regions, including the brainstem, limbic system, and cortical networks involved in cognition and emotional regulation [7,14]. In parallel, epidemiological investigations have reported increased risks of neurological and neuropsychiatric disorders following SARS-CoV-2 infection, reinforcing the hypothesis that COVID-19 may induce long-lasting effects on the nervous system [15].

Emerging evidence suggests that persistent cognitive dysfunction in Long COVID may reflect alterations in large-scale neural networks involved in attention, executive function, and memory processing [16,17,18]. These findings support the interpretation of NeuroCOVID as a multidimensional condition involving neurological, neuropsychological, and functional domains rather than a collection of isolated symptoms.

Although the underlying mechanisms remain incompletely understood, persistent neuroinflammation, immune dysregulation, endothelial dysfunction, and autonomic imbalance have been proposed as potential contributors to persistent NeuroCOVID manifestations [3,19,20].

Beyond symptom persistence, increasing attention has been directed toward the functional consequences of Long COVID. Several studies have shown that persistent neurological, cognitive, and neuropsychological manifestations may substantially impair activities of daily living, work ability, social participation, and health-related quality of life. Functional limitations appear to result from the cumulative burden of multiple persistent symptoms rather than from isolated manifestations, highlighting the need for multidimensional patient assessment. However, relatively few studies have specifically examined whether the overall burden of patient-reported NeuroCOVID symptoms is independently associated with functional impairment, leaving an important gap in current evidence.

Despite the growing body of evidence regarding NeuroCOVID, important knowledge gaps remain. Most studies have focused primarily on symptom prevalence and clinical characterization, whereas less attention has been devoted to understanding the real-world functional consequences of neurological symptom burden [5,21]. In particular, limited evidence is available regarding the relationship between persistent neurocognitive symptoms and restrictions in work activities, education, social participation, self-care, and daily functioning. From a public health perspective, understanding this relationship is essential for identifying individuals at risk of long-term disability and for developing targeted rehabilitation and support strategies.

In Italy, our research group previously developed and psychometrically validated a questionnaire specifically designed to assess post-COVID manifestations, demonstrating excellent reliability and internal consistency [22]. Subsequently, using the same cohort, we described the clinical and symptom profiles of Long COVID survivors, identifying fatigue, weakness, musculoskeletal pain, and headache as the most common persistent manifestations [23,24]. However, neither study specifically investigated the neurological symptom burden nor its association with functional impairment.

Importantly, although based on the same cohort, the present study addresses a distinct research question that has not been examined in our previous publications. While previous analyses focused on questionnaire validation and the overall characterization of Long COVID symptom profiles, the current investigation introduces a NeuroCOVID-specific analytical framework by quantifying neurological, neurocognitive, neurosensory, autonomic, and neuropsychological symptom burden through a composite score and examining its relationship with functional outcomes. Therefore, all analyses regarding the NeuroCOVID Burden Score, Functional Impairment Score association, symptom-specific correlations, and multivariable predictive models represent original analyses not previously reported.

Secondary analyses of well-characterized cohorts allow investigators to address new clinically relevant research questions without additional participant burden while maximizing the scientific value of existing datasets.

Therefore, the present study was designed to address this gap. Using a secondary analysis of a cohort of Italian adults with Long COVID, we aimed to quantify NeuroCOVID burden and evaluate its association with functional limitations in daily life [25,26]. We hypothesized that a higher burden of persistent neurological and neuropsychological symptoms would be independently associated with greater functional impairment, even after accounting for demographic and clinical characteristics.

## 2. Materials and Methods

### 2.1. Study Design and Reporting Guideline

This study represents a secondary cross-sectional analysis of data collected through a validated questionnaire designed to assess post-COVID-19 condition (Long COVID) among adults residing in Italy. The study was reported according to the Strengthening the Reporting of Observational Studies in Epidemiology (STROBE) guidelines for observational studies [27]. The completed STROBE checklist is provided as Supplementary Material S1. The present investigation focused specifically on the neurological and neuropsychological dimensions of Long COVID (NeuroCOVID) and their association with functional impairment in daily life activities.

### 2.2. Study Population and Setting

The study included 250 adults aged 18 years or older with a previous confirmed SARS-CoV-2 infection and persistent symptoms compatible with post-COVID-19 condition. Participants were recruited between February and April 2025 through online dissemination and community-based invitations. Data collection was conducted over a three-month period (February–April 2025), after which the database was checked for completeness and prepared for the present secondary analysis. No formal a priori sample size calculation was performed for this secondary analysis, as the present investigation used an existing dataset derived from the validation cohort.

Inclusion Criteria: Participants were eligible if they were aged ≥18 years; reported a previous SARS-CoV-2 infection; experienced persistent symptoms lasting at least 12 weeks after the acute infection; provided informed consent.

Exclusion Criteria: Participants were excluded if they had severe pre-existing neurological disorders that could interfere with symptom interpretation; were unable to complete the questionnaire because of cognitive or language barriers; provided incomplete questionnaires preventing score calculation.

A formal participant flow diagram was not developed because the present study represents a secondary analysis of a completed cross-sectional dataset and all eligible participants were included in the final analytical sample.

The original dataset was screened for completeness before analysis. Only participants who completed all questionnaire sections required for sociodemographic characterization, NeuroCOVID Burden Score calculation, and Functional Impairment Score calculation were retained. Therefore, the final analytical sample consisted exclusively of complete cases.

### 2.3. Questionnaire

Data were collected using a previously validated questionnaire specifically developed to assess post-COVID-19 manifestations in the Italian population.

The questionnaire consisted of four main sections: (1) sociodemographic and clinical characteristics (age, sex, vaccination status, hospitalization, and acute COVID-19 severity); (2) patient-reported persistent NeuroCOVID manifestations, including neurological, neurocognitive, neuropsychological, neurosensory, and autonomic symptoms; (3) functional impairment across ten domains of daily life, including work, education, mobility, self-care, household activities, social participation, and interpersonal relationships; and (4) general health information. All symptom and functional impairment items were assessed using a five-point Likert scale ranging from 1 (no impairment) to 5 (very severe impairment), with higher scores indicating greater symptom burden or functional limitation.

No separate pilot study was conducted for the present secondary analysis because no new questionnaire was administered and no new data were collected. The present analysis used selected items from the previously validated questionnaire to construct two exploratory composite scores addressing a distinct research question.

### 2.4. Variables

NeuroCOVID Variables: Persistent neurological and neuropsychological symptoms were evaluated using Likert-scale items ranging from 1 (not at all) to 5 (extremely).

The following symptoms were included in the NeuroCOVID domain: headache, concentration difficulties, memory problems, olfactory dysfunction, taste/swallowing dysfunction, tinnitus/ear pain/dysphonia, sleep disorders, mood disorders, depression/anxiety, and tachycardia.

Functional Variables: Functional impairment was assessed through participant-reported limitations in work activities; educational activities; domestic activities; recreational activities; mobility; social relationships; health management; self-care.

Higher scores reflected greater levels of impairment.

### 2.5. NeuroCOVID Burden Score

The NeuroCOVID Burden Score was derived from ten patient-reported symptom items: headache, concentration difficulties, memory problems, olfactory dysfunction, taste/swallowing dysfunction, tinnitus/ear pain/dysphonia, sleep disorders, mood disorders, depression/anxiety, and tachycardia. Each item was rated on a five-point Likert scale from 1 to 5, with higher values indicating greater symptom severity. For each participant, the score was calculated as the arithmetic mean of the ten item scores:

NeuroCOVID Burden Score = (sum of the ten symptom-item scores)/10.

The resulting score therefore retained the original 1–5 metric, with higher values indicating greater overall patient-reported NeuroCOVID burden. All items were equally weighted because no validated or clinically established weighting algorithm is currently available for these symptom domains. Internal consistency was evaluated using Cronbach’s alpha. The score was developed as an exploratory analytical composite for the present secondary analysis and should not be interpreted as a validated diagnostic or clinical decision-making instrument.

### 2.6. Functional Impairment Score

The Functional Impairment Score was derived from eight patient-reported domains: work activities, educational activities, domestic activities, recreational activities, mobility, social relationships, health management, and self-care. Each domain was rated on a five-point Likert scale from 1 to 5, with higher values indicating greater functional limitation. For each participant, the score was calculated as the arithmetic mean of the eight domain scores:

Functional Impairment Score = (sum of the eight functional-domain scores)/8.

The resulting score retained the original 1–5 metric, with higher values indicating greater overall functional impairment. All domains were equally weighted because no externally validated weighting system was available and the analytical objective was to summarize global patient-reported functional limitation. Internal consistency was assessed using Cronbach’s alpha. As with the NeuroCOVID Burden Score, this composite should be regarded as an exploratory analytical measure rather than a fully validated clinical scale.

### 2.7. Statistical Analysis

Continuous variables were summarized using means and standard deviations (SD), whereas categorical variables were described using frequencies and percentages. The internal consistency of the NeuroCOVID Burden Score and Functional Impairment Score was evaluated using Cronbach’s alpha. Pearson correlation coefficients were calculated to investigate the relationship between NeuroCOVID burden and functional impairment, and Spearman correlation coefficients were additionally computed as a sensitivity analysis.

To identify independent predictors of functional impairment, multivariable linear regression models were developed using the Functional Impairment Score as the dependent variable. Independent variables included NeuroCOVID Burden Score, age, sex, comorbidities, reinfection history, vaccination status, and self-reported severity of acute COVID-19. Results are presented as unstandardized regression coefficients (B), standardized regression coefficients (β), 95% confidence intervals (95% CIs), *p*-values, and coefficients of determination (R^2^), where appropriate. Statistical significance was established at *p* < 0.05. All statistical analyses were performed using R statistical software (version 4.3.1; R Foundation for Statistical Computing, Vienna, Austria). Statistical significance was established at *p* < 0.05.

Composite scores were calculated for participants with available responses for the corresponding items. Regression assumptions were assessed by examining linearity, residual distribution, homoscedasticity, influential observations, and multicollinearity using variance inflation factors. Potential sources of bias, including self-reporting bias, recall bias, and selection bias related to voluntary participation, were considered during interpretation of the findings.

### 2.8. Ethical Considerations

The study was conducted in compliance with the ethical standards set by the Declaration of Helsinki and with EU Regulation 2016/679 (GDPR). Ethical approval was granted by the Territorial Ethics Committee Campania 2 (protocol no. 2025/3339, 5 February 2025), which ensured that all procedures were carried out in a manner prioritizing participants’ safety, privacy, and autonomy. Written informed consent was obtained from all participants prior to inclusion in the study, after receiving clear information about objectives, confidentiality, and voluntary participation. To ensure data confidentiality, all questionnaire responses were collected anonymously and stored in a password-protected electronic database accessible only to the research team. No directly identifiable personal information was included in the analytical dataset. Data processing complied with the General Data Protection Regulation (EU Regulation 2016/679, GDPR), and all analyses were performed using anonymized data.

## 3. Results

### 3.1. Participant Characteristics

The final analytical sample included 250 participants. The mean age was 33.19 years (SD 12.91; median 29; range 18–73), and 159 participants were female (63.6%). Overall, 140 participants (56.0%) were aged 30 years or younger. Pre-existing comorbidities were reported by 11 participants (4.4%). Hospitalization during acute COVID-19 was uncommon, being reported by only one participant (0.4%). Reinfection was reported by 87 participants (34.8%). Most participants reported a mild-to-moderate acute disease course, with severity levels 2 and 3 each reported by 100 participants (40.0%). The final analytical sample included 250 participants with complete data available for all variables included in the analyses. Overall, participants were predominantly young adults, most were female, and hospitalization during acute COVID-19 was uncommon. Most participants reported a mild-to-moderate acute disease course. Detailed sociodemographic and clinical characteristics are presented in Table 1.

### 3.2. NeuroCOVID Symptom Profile

Persistent neurological, neurocognitive, neurosensory, autonomic, and neuropsychological manifestations were reported with heterogeneous severity across symptom domains (Table 2). Headache, mood disorders, and concentration difficulties represented the most prominent patient-reported NeuroCOVID manifestations, whereas tinnitus/ear pain/dysphonia and tachycardia generally showed lower symptom severity. Overall, neuropsychological and cognitive symptoms constituted the predominant components of the NeuroCOVID profile.

Using a threshold of moderate or greater intensity (Likert score ≥ 3), headache, mood disorders, and concentration difficulties were also the most frequently reported NeuroCOVID manifestations. Detailed prevalence and severity estimates for each symptom domain are presented in Table 2.

### 3.3. Functional Impairment Profile

Functional impairment was heterogeneous across the evaluated domains (Table 3). The greatest limitations were observed in study- and work-related activities, followed by recreational and domestic activities, whereas mobility and self-care were comparatively less affected.

Moderate-to-severe functional impairment (Likert score ≥ 3) was reported most frequently for study-, work-, and domestic activities, while self-care was the least affected domain. Detailed descriptive statistics and prevalence estimates for each functional domain are presented in Table 3.

### 3.4. Reliability of Composite Scores

**Figure 1:**
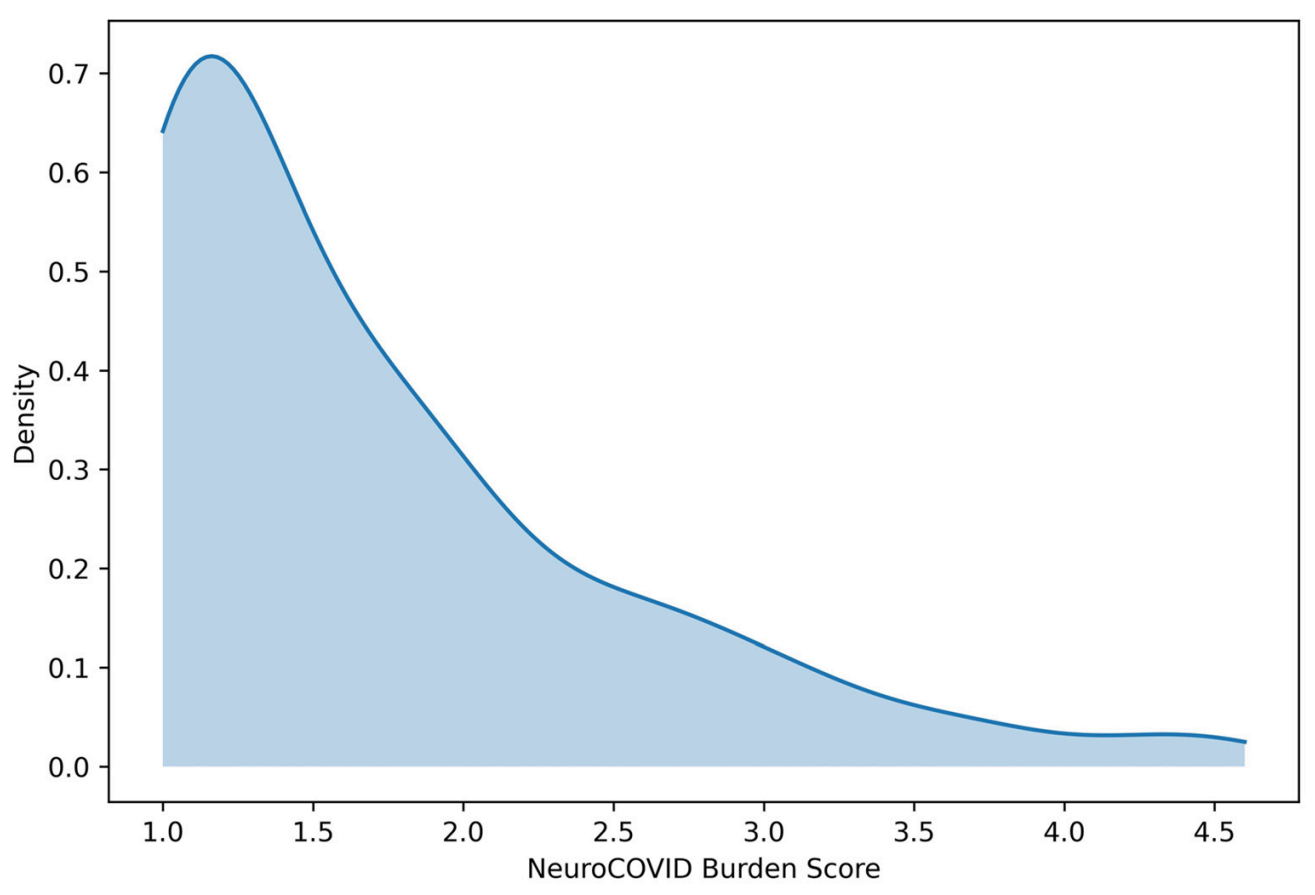
Density distribution of NeuroCOVID Burden Scores among Long COVID survivors. Kernel density plot illustrating the distribution pattern of patient-reported NeuroCOVID Burden Scores in the study population (*N* = 250). Higher scores indicate greater neurological, neurocognitive, neurosensory, autonomic, and neuropsychological symptom burden.

Both composite scores demonstrated high internal consistency, supporting their use as exploratory analytical measures in the subsequent analyses (Table 4). The distribution of NeuroCOVID Burden Scores is illustrated in Figure 1. Reliability estimates and correlation analyses are summarized in Table 4.

### 3.5. Association Between NeuroCOVID Burden and Functional Impairment

**Figure 2:**
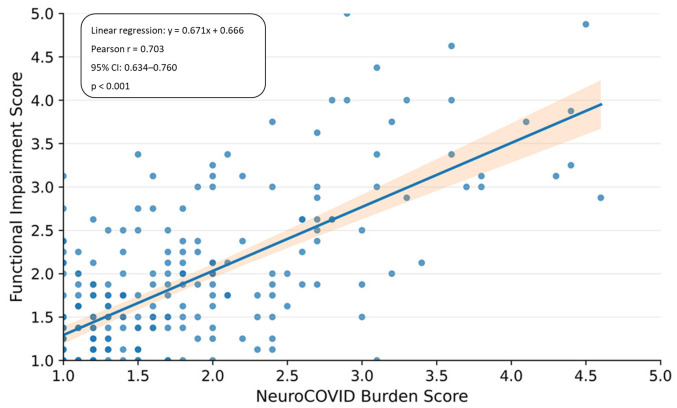
Association between NeuroCOVID Burden Score and Functional Impairment Score. Scatter plot illustrating the relationship between patient-reported NeuroCOVID Burden Score and Functional Impairment Score. The solid line represents the fitted linear regression model, whereas the shaded area indicates the 95% confidence interval around the regression estimate. The regression equation, Pearson correlation coefficient (r), corresponding 95% confidence interval, and statistical significance are reported within the figure.

A strong positive association was observed between NeuroCOVID Burden Score and Functional Impairment Score, and this relationship remained consistent across both Pearson and Spearman correlation analyses (Table 4). The linear association between the two composite scores is illustrated in Figure 2.

**Figure 3:**
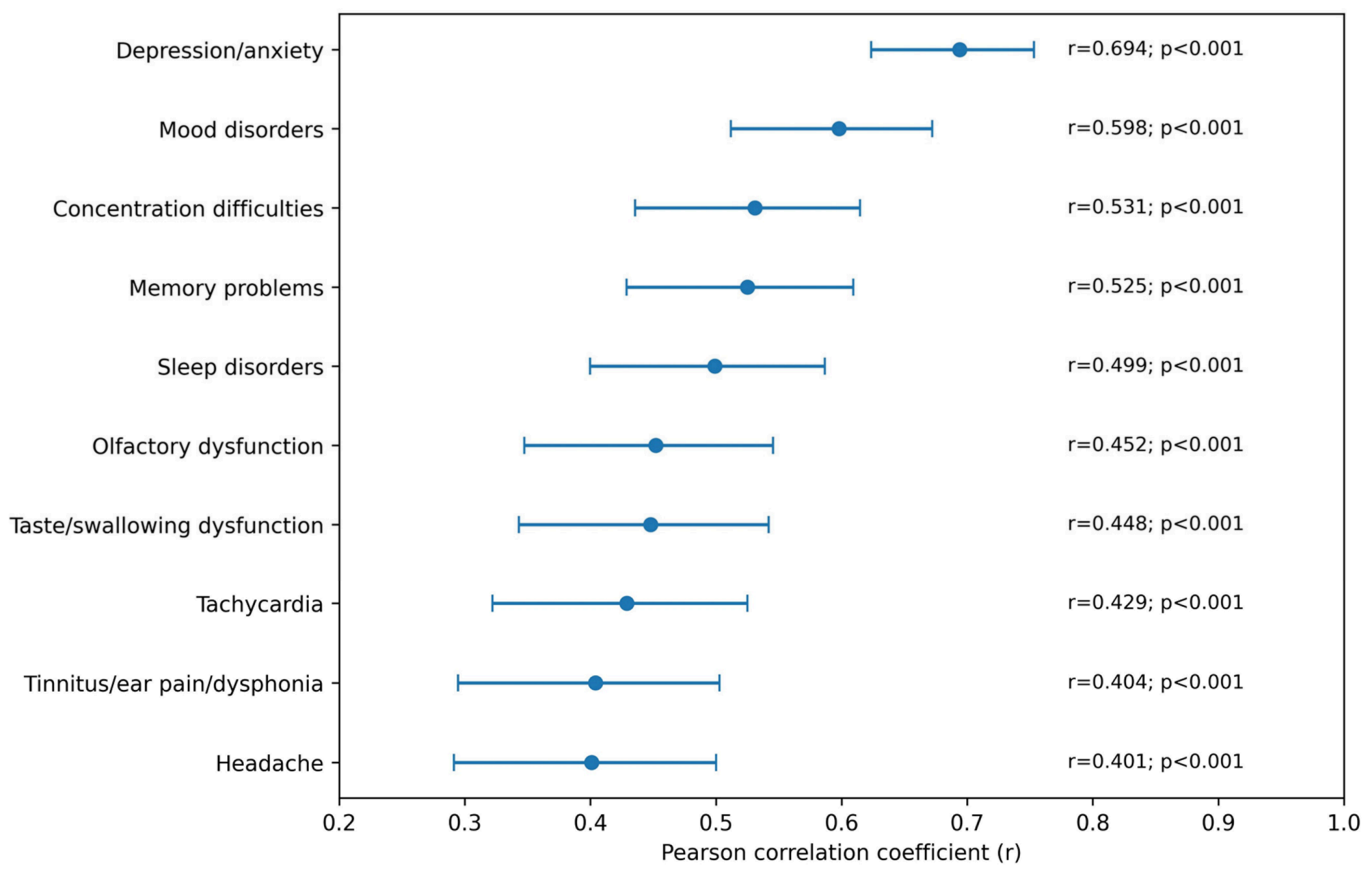
Correlations between individual NeuroCOVID symptoms and Functional Impairment Score. Forest plot illustrating Pearson correlation coefficients between individual patient-reported NeuroCOVID symptoms and Functional Impairment Score. Points represent Pearson correlation coefficients (r), whereas horizontal bars indicate corresponding 95% confidence intervals. Statistical significance values are reported for each symptom-level association.

Individual NeuroCOVID manifestations showed heterogeneous associations with functional impairment (Figure 3). Neuropsychological and cognitive symptoms—particularly depression/anxiety, mood disorders, concentration difficulties, memory problems, and sleep disorders—demonstrated the strongest correlations, whereas headache and neurosensory or autonomic manifestations showed comparatively weaker associations. Complete correlation coefficients, 95% confidence intervals, and *p*-values are reported in Figure 3.

### 3.6. Multivariable Regression Analysis

In the multivariable linear regression model, NeuroCOVID Burden Score remained independently associated with functional impairment after adjustment for age, sex, comorbidities, hospitalization, reinfection, vaccination status, and acute COVID-19 severity (Table 5). NeuroCOVID Burden Score represented the strongest independent predictor of functional impairment in the adjusted model, whereas acute COVID-19 severity showed a smaller but statistically significant independent association. The remaining covariates were not significantly associated with the outcome. The multivariable model explained 52.5% of the variance in functional impairment (R^2^ = 0.525).

## 4. Discussion

### 4.1. Main Findings

This secondary analysis showed that greater patient-reported NeuroCOVID burden was strongly associated with higher functional impairment among adults with Long COVID. The association remained significant after adjustment for the demographic and clinical variables available in the dataset, while acute COVID-19 severity showed a smaller independent association with functional impairment.

These findings suggest that the cumulative burden of neurological, neurocognitive, neurosensory, autonomic, and neuropsychological symptoms may provide clinically relevant information beyond the assessment of individual manifestations. However, given the cross-sectional design, the observed relationships should be interpreted as associations rather than evidence of causality or temporal direction.

Importantly, the clinical relevance of this association is reflected in the breadth of the functional domains involved. Participants with a greater cumulative NeuroCOVID burden reported more pronounced limitations across multiple aspects of everyday functioning, including work, educational activities, domestic tasks, recreation, social relationships, and health management. Therefore, the observed association extends beyond statistical significance and indicates that persistent patient-reported NeuroCOVID manifestations may have meaningful implications for patients’ independence, participation in daily life, and overall functional well-being.

### 4.2. Interpretation in Relation to Previous Evidence

The strong association observed in this study between NeuroCOVID burden and functional impairment is particularly relevant because existing evidence, including published reviews, has predominantly focused on symptom prevalence rather than functional consequences. The present findings extend previous evidence by shifting attention from symptom prevalence to the functional consequences of cumulative patient-reported NeuroCOVID burden. Previous large observational studies, together with systematic review evidence, have consistently documented persistent deficits in attention, executive function, memory, and processing speed, which may interfere with occupational performance, educational activities, social participation, and quality of life [9,10,11,12].

Although previous studies have reported associations between persistent neurological symptoms and reduced functional status after COVID-19, these investigations have generally evaluated individual symptoms or isolated cognitive outcomes. In contrast, the present study examined the cumulative burden of neurological, neurocognitive, neuropsychological, neurosensory, and autonomic manifestations using a composite NeuroCOVID Burden Score and assessed its independent association with multidimensional functional impairment after adjustment for relevant demographic and clinical variables.

Our results support this broader functional perspective, indicating that the combined burden of cognitive, psychological, sensory, autonomic, and sleep-related symptoms is closely associated with limitations across multiple domains of daily life.

Unlike our previous validation and descriptive analyses, the present study specifically examined the association between a NeuroCOVID-focused composite burden measure and functional impairment. The symptom-specific correlations and adjusted regression analyses therefore provide a distinct analytical contribution that was not reported in the earlier publications. These findings therefore complement, rather than duplicate, previous evidence by providing a multidimensional analytical framework focused on overall patient-reported NeuroCOVID burden instead of isolated neurological manifestations, thereby offering additional insight into the relationship between symptom burden and functional limitations in daily life.

### 4.3. Possible Explanatory Mechanisms

Several mechanisms may explain the association between NeuroCOVID burden and functional impairment. Persistent neuroinflammation, immune dysregulation, endothelial dysfunction, microvascular injury, autonomic nervous system imbalance, viral persistence, and mitochondrial dysfunction have all been proposed as potential contributors to Long COVID symptoms [2,3,13,14,15]. These mechanisms may affect central and peripheral nervous system functioning, resulting in cognitive impairment, altered sleep regulation, mood disturbances, dysautonomia, and reduced tolerance to physical or cognitive effort.

Sleep disturbances and olfactory dysfunction may represent clinically relevant components of the NeuroCOVID phenotype. Beyond their individual impact, these alterations may interact with mechanisms involved in cognitive processing, neural plasticity, and the continuous updating of internal models required for adaptive behavior [16,17].

Similar pathways have been implicated in early neurodegenerative processes, including cognitive decline associated with Alzheimer’s disease. Although Long COVID and neurodegenerative diseases represent distinct clinical entities, overlapping mechanisms involving neuroinflammation, network dysfunction, altered sensory processing, and reduced cognitive resilience may partially explain the relationship between NeuroCOVID symptoms and functional impairment observed in our cohort [16,17,18].

Because no neuroimaging, biomarker, or neurophysiological assessments were performed, the biological mechanisms discussed above remain speculative and are intended only to provide a plausible interpretative framework for the observed associations. Future longitudinal and mechanistic studies are required to directly investigate these pathways.

The association between depression/anxiety, mood disorders, cognitive symptoms, and functional impairment should not be interpreted as evidence that Long COVID is primarily psychological. Rather, these dimensions likely interact within a biopsychosocial and neuroimmune framework. Persistent symptoms can generate functional limitation, while functional limitation may worsen psychological distress, sleep quality, and perceived health. Because of the cross-sectional design, the directionality of these relationships cannot be established in the present study.

### 4.4. Clinical and Public Health Implications

These findings have practical implications for clinical assessment and follow-up. Our findings suggest that comprehensive clinical assessment of individuals with Long COVID may benefit from including neurocognitive, neuropsychological, neurosensory, and autonomic domains, particularly in patients reporting difficulties returning to work, study, social life, or usual daily activities. However, prospective studies are needed to determine the clinical utility and cost-effectiveness of routine NeuroCOVID screening. The use of structured tools may support the early identification of individuals with a higher patient-reported NeuroCOVID burden who may be at increased risk of functional impairment.

Although requiring external validation, the NeuroCOVID Burden Score may represent a promising exploratory screening tool for use within multidisciplinary Long COVID pathways. However, its clinical implementation should be confirmed in prospective validation studies before routine use can be recommended. Accordingly, its potential clinical utility should be interpreted cautiously until further psychometric and external validation studies become available. Different symptom clusters may support individualized rehabilitation strategies: cognitive symptoms may require neuropsychological rehabilitation approaches, sleep and mood disturbances may benefit from integrated psychological and behavioral interventions, whereas autonomic symptoms may require specific clinical evaluation and targeted rehabilitation programs.

From a public health perspective, our findings suggest that multidisciplinary post-COVID care pathways may be appropriate for individuals with a high NeuroCOVID symptom burden. Nevertheless, the effectiveness of such multidisciplinary approaches was not evaluated in the present study and should be investigated in future prospective research. In particular, nursing and community-based care may play a relevant role in symptom monitoring, functional assessment, patient education, self-management support, and referral to specialized services when needed. Given the potential impact of NeuroCOVID on work capacity and social participation, functional outcomes should be considered central endpoints in future Long COVID research and care planning. The relevance of functional outcomes is consistent with previous evidence showing that health status, psychosocial well-being, and functional capacity may substantially influence participation and performance in daily activities and social roles [28].

### 4.5. Strengths and Limitations

This study has several strengths. First, it used individual-level data from a cohort assessed with a previously validated Long COVID questionnaire. Although the NeuroCOVID Burden Score demonstrated excellent internal consistency, this finding alone does not establish its overall psychometric validity. The score was developed as an exploratory analytical composite for the present secondary analysis and therefore requires external validation, including assessment of its construct validity, dimensionality, responsiveness, test–retest reliability, predictive performance, and external validity in independent Long COVID cohorts before any routine clinical application. Second, the analysis focused on a clearly defined and clinically relevant research question: the association between NeuroCOVID burden and functional impairment. Third, both the NeuroCOVID Burden Score and the Functional Impairment Score showed high internal consistency. Fourth, the multivariable model adjusted for several demographic and clinical factors, including age, sex, comorbidities, reinfection, vaccination status, and acute disease severity. Finally, the study contributes data from an Italian cohort, adding evidence from a context that remains less represented than large international hospital-based datasets.

Several limitations should also be acknowledged. First, the cross-sectional design precludes causal inference and does not allow conclusions regarding the temporal relationship between NeuroCOVID burden and functional impairment. Although NeuroCOVID burden was strongly associated with functional impairment, it is not possible to determine whether neurological symptoms contributed to functional limitations, whether functional impairment influenced symptom perception, or whether both were driven by shared underlying mechanisms.

Second, participants were recruited through voluntary online participation, which may have introduced selection bias by preferentially including younger, digitally engaged individuals and those experiencing persistent symptoms. Consequently, the findings may not be representative of the entire Italian Long COVID population. Moreover, the study cohort consisted predominantly of relatively young, community-dwelling adults with a low prevalence of severe acute COVID-19, hospitalization, and major comorbidities. Therefore, the generalizability of the findings to older adults, hospitalized patients, or individuals with more clinically complex Long COVID should be interpreted with caution.

Third, although the multivariable regression model adjusted for several demographic and clinical variables, residual confounding cannot be excluded because potentially relevant factors—including educational level, socioeconomic status, occupational status, pre-existing psychiatric disorders, medication use, rehabilitation interventions, and pre-COVID sleep quality—were not available in the original dataset.

Fourth, both the NeuroCOVID Burden Score and the Functional Impairment Score were derived from patient-reported questionnaire responses. Although patient-reported outcomes are essential for capturing the subjective experience of Long COVID, they remain susceptible to recall bias, reporting bias, symptom misclassification, and subjective interpretation of Likert-scale items. Furthermore, no standardized neuropsychological testing, neurological examination, neuroimaging, or biological biomarkers were available to corroborate the reported symptoms. Consequently, the NeuroCOVID Burden Score reflects perceived rather than objectively confirmed neurological burden, and subjective cognitive complaints may not fully correspond to objectively measured cognitive impairment.

Finally, although both composite scores demonstrated excellent internal consistency, Cronbach’s alpha alone does not establish construct validity, dimensionality, criterion validity, responsiveness, test–retest reliability, or external validity. Accordingly, these scores should be considered exploratory analytical measures and require further psychometric evaluation in independent populations before any clinical application. Future studies should integrate patient-reported outcomes with standardized neuropsychological assessments, neurological examination, neuroimaging, and biological biomarkers to provide a more comprehensive characterization of NeuroCOVID and its relationship with functional impairment.

### 4.6. Future Research

Future studies should validate the NeuroCOVID Burden Score in larger and more diverse populations, ideally using longitudinal designs. Further research should evaluate additional psychometric properties beyond internal consistency, including factorial dimensionality, construct validity, test–retest reliability, and responsiveness to longitudinal clinical changes. Such analyses will be essential to determine whether the score can reliably capture NeuroCOVID trajectories, disease evolution, and treatment-related improvements over time. Combining patient-reported symptoms with objective cognitive testing, neurological examination, digital functional assessment, and biomarkers may help clarify the mechanisms linking NeuroCOVID to disability. Longitudinal analyses are also needed to determine whether NeuroCOVID burden predicts persistence, recovery, relapse, or worsening of functional impairment over time. Finally, interventional studies should evaluate whether multidisciplinary rehabilitation and nursing-led follow-up can reduce NeuroCOVID burden and improve daily functioning.

## 5. Conclusions

In this secondary analysis of an Italian Long COVID cohort, persistent NeuroCOVID burden was strongly and independently associated with functional impairment across multiple domains of daily life. Participants reporting higher levels of neurological, neurocognitive, neurosensory, autonomic, and neuropsychological symptoms experienced greater limitations in work, study, domestic activities, social participation, mobility, health management, and self-care.

Among the variables examined, NeuroCOVID burden was the factor most strongly associated with functional impairment in the adjusted model, accounting for a substantial proportion of the observed variability even after adjustment for demographic and clinical characteristics. These findings suggest that persistent patient-reported NeuroCOVID manifestations are closely associated with functional limitations across multiple domains of everyday life. However, the cross-sectional design does not allow conclusions regarding causality or temporal direction.

The study contributes to the growing evidence that patient-reported NeuroCOVID manifestations are clinically relevant because of their association with functional limitations. However, the present findings do not establish the clinical utility of routine NeuroCOVID assessment or multidisciplinary management pathways. Rather, they identify patient-reported NeuroCOVID burden as a potential marker of functional impairment that warrants further investigation. The clinical utility of the NeuroCOVID Burden Score and its possible role in comprehensive Long COVID assessment require external validation and prospective evaluation before any recommendation for routine clinical practice can be made. Accordingly, the NeuroCOVID Burden Score should be considered an exploratory analytical measure rather than a validated clinical instrument for individual patient assessment or clinical decision-making.

Future multicenter prospective longitudinal studies are needed to validate the NeuroCOVID Burden Score, better define the temporal relationship between NeuroCOVID manifestations and functional impairment, and evaluate their evolution over time. Because the present study was conducted in a predominantly young, community-based Italian cohort with very few hospitalized participants, further research should also examine the generalizability of these findings in older adults, hospitalized patients, and more clinically diverse Long COVID populations. The integration of standardized neuropsychological assessments, comprehensive neurological examination, neuroimaging, and biological biomarkers may help validate patient-reported outcomes and improve understanding of the biological mechanisms underlying persistent NeuroCOVID. Future interventional studies should also determine whether targeted multidisciplinary interventions improve long-term functional outcomes.

## Figures and Tables

**Table 1:** Sociodemographic and Clinical Characteristics of the Study Population (*N* = 250).

Variable	*n* (%) or Mean ± SD
**Age (years)**	
Mean ± SD	33.19 ± 12.91
Median (IQR)	29 (23–40)
Range	18–73
**Age group**	
≤30 years	140 (56.0)
>30 years	110 (44.0)
**Sex**	
Female	159 (63.6)
Male	91 (36.4)
**Smoking status**	
Never smoker	114 (45.6)
Former smoker	40 (16.0)
Occasional smoker	19 (7.6)
Current smoker	77 (30.8)
**Comorbidities**	
Yes	11 (4.4)
No	239 (95.6)
**Hospitalization during acute COVID-19**	
Yes	1 (0.4)
No	249 (99.6)
**COVID-19 reinfection**	
Yes	87 (34.8)
No	163 (65.2)
**Vaccination status**	
1 dose	25 (10.0)
2 doses	101 (40.4)
3 doses	122 (48.8)
4 doses	2 (0.8)
**Acute COVID-19 severity**	
1 (Asymptomatic)	14 (5.6)
2 (Mild)	100 (40.0)
3 (Moderate)	100 (40.0)
4 (Severe)	31 (12.4)
5 (Critical)	5 (2.0)

**Abbreviations:** SD, standard deviation; IQR, interquartile range.

**Table 2:** Persistent NeuroCOVID Symptom Burden Among Long COVID Survivors (*N* = 250).

Symptom Domain	Mean ± SD	Moderate-to-Severe *n* (%)
Headache	1.94 ± 1.26	74 (29.6)
Mood disorders	1.89 ± 1.21	69 (27.6)
Concentration difficulties	1.84 ± 1.20	66 (26.4)
Olfactory dysfunction	1.70 ± 1.19	52 (20.8)
Depression/Anxiety	1.70 ± 1.13	49 (19.6)
Taste/swallowing dysfunction	1.68 ± 1.16	44 (17.6)
Memory problems	1.67 ± 1.09	54 (21.6)
Sleep disorders	1.65 ± 1.13	47 (18.8)
Tinnitus/Ear pain/Dysphonia	1.59 ± 1.00	44 (17.6)
Tachycardia	1.52 ± 1.03	38 (15.2)

**Note:** Moderate-to-severe symptoms were defined as Likert-scale scores ≥ 3.

**Table 3:** Functional Impairment Across Daily Life Domains (*N* = 250).

Functional Domain	Mean ± SD	Moderate-to-Severe *n* (%)
Study activities	2.12 ± 1.27	85 (34.0)
Work activities	2.01 ± 1.11	75 (30.0)
Recreational activities	1.94 ± 1.22	67 (26.8)
Domestic activities	1.91 ± 1.07	69 (27.6)
Social relationships	1.82 ± 1.20	64 (25.6)
Health management	1.75 ± 1.07	56 (22.4)
Mobility	1.62 ± 1.04	45 (18.0)
Self-care	1.42 ± 0.85	29 (11.6)

**Note:** Moderate-to-severe impairment was defined as Likert-scale scores ≥ 3.

**Table 4:** Reliability and Correlation Analyses.

Analysis	Estimate	95% CI	*p*-Value
NeuroCOVID Burden Score (Cronbach’s α)	0.890	—	—
Functional Impairment Score (Cronbach’s α)	0.898	—	—
Pearson correlation (NeuroCOVID Burden vs. Functional Impairment)	0.703	0.634–0.760	<0.001
Spearman correlation (NeuroCOVID Burden vs. Functional Impairment)	0.652	—	<0.001

**Abbreviations:** CI, confidence interval.

**Table 5:** Multivariable Linear Regression Analysis of Functional Impairment.

Dependent Variable: Functional Impairment Score				
Predictor	Unstandardized B	Standardized β	95% CI	*p*-Value
NeuroCOVID Burden Score	0.671	0.671	0.566–0.776	<0.001
Acute COVID-19 severity	0.156	0.156	0.055–0.257	0.003
Age (years)	−0.005	−0.005	−0.011–0.001	0.118
Female sex	−0.130	−0.130	−0.286–0.027	0.104
Presence of comorbidities	0.242	0.242	−0.130–0.615	0.201
COVID-19 reinfection	−0.036	−0.058	−0.216–0.101	0.474
Number of vaccine doses received	0.671	−0.036	−0.147–0.075	0.523
**Model Statistics**				
**Statistic**		**Value**		
R^2^		0.525		
Overall model *p*-value		<0.001		

**Abbreviations:** CI, confidence interval.

## Data Availability

The data underlying the findings of this study are available from the corresponding author upon reasonable request. The study protocol and related materials are available on the Open Science Framework (OSF) at DOI: 10.17605/OSF.IO/XRTSE.

## Supplementary Materials

The following supporting information can be downloaded at: https://www.mdpi.com/article/10.3390/jcm15156027/s1, Supplementary Material S1: STROBE Statement—Checklist of items that should be included in reports of cross-sectional studies.
