# Supplementary material for: NeuroCOVID Burden and Functional Impairment in Adults with Long COVID: A Secondary Analysis of an Italian Cohort
Source: J Clin Med. 2026 Aug 3;15(15):6027. doi: 10.3390/jcm15156027 (PMC13467503; doi:10.3390/jcm15156027)
Supplement: Supplementary file 1 [file jcm-15-06027-s001.zip › jcm-4437229-supplementary.pdf]

STROBE Statement—Checklist of items that should be included in reports of cross-sectional studies

NeuroCOVID Burden and Functional Impairment in Adults

Item	Recommendation	Reported	Location in Manuscript
**1a**	Indicate study design in title/abstract	Yes	Title; Abstract Methods
**1b**	Informative and balanced abstract	Yes	Abstract
**2**	Scientific background and rationale	Yes	Introduction paragraphs 1–11
**3**	Objectives and hypotheses	Yes	End of Introduction
**4**	Study design	Yes	Section 2.1 Study Design and Reporting Guideline
**5**	Setting, locations and dates	Yes	Section 2.2 Study Population and Setting
**6a**	Eligibility criteria and participant selection	Yes	Section 2.2 Inclusion and Exclusion Criteria
**6b**	Matching criteria (if applicable)	N/A	No matched design
**7**	Definition of outcomes, exposures, predictors and confounders	Yes	Sections 2.4–2.6
**8**	Data sources and measurements	Yes	Section 2.3 Questionnaire; Section 2.4 Variables
**9**	Address potential sources of bias	Yes	Section 2.2 Study Population and Setting
**10**	Explain study size	Yes	Cohort size reported; no formal sample size calculation
**11**	Handling of quantitative variables	Yes	Sections 2.5–2.7
**12a**	Statistical methods	Yes	Section 2.7 Statistical Analysis
**12b**	Subgroup/interactions	No	Not performed
**12c**	Missing data	Yes	Section 2.7 Statistical Analysis
**12d**	Loss to follow-up	N/A	Cross-sectional study
**12e**	Sensitivity analyses	Yes	Spearman correlation analysis reported
**13a**	Number of participants at each stage	Yes	Results 3.1 Participant Characteristics
**13b**	Reasons for non-participation	No	Not available
**13c**	Flow diagram	No	Not included
**14a**	Participant characteristics	Yes	Table 1; Results 3.1
**14b**	Missing data by variable	Partial	Addressed in Methods but not tabulated
**14c**	Follow-up time	N/A	Cross-sectional study
**15**	Outcome data	Yes	Tables 2–5; Results sections 3.2–3.6
**16a**	Unadjusted and adjusted estimates	Yes	Correlation analysis; Multivariable regression model
**16b**	Category boundaries reported	Yes	Likert scale categories and severity thresholds reported
**16c**	Absolute risk translation	N/A	Not applicable
**17**	Other analyses	Yes	Spearman sensitivity analysis; symptom-specific correlations
**18**	Key results summarized	Yes	Discussion 4.1 Main Findings
**19**	Limitations discussed	Yes	Section 4.5 Strengths and Limitations
**20**	Interpretation of results	Yes	Sections 4.2–4.4 and Conclusions
**21**	Generalisability	Yes	Section 4.6 Limitations
**22**	Funding source	Yes	Funding Statement

Note: An Explanation and Elaboration article discusses each checklist item and gives methodological background and published examples of transparent reporting. The STROBE checklist is best used in conjunction with this article (freely available on the Web

sites of PLoS Medicine at http://www.plosmedicine.org/, Annals of Internal Medicine at http://www.annals.org/, and Epidemiology at http://www.epidem.com/). Information on the STROBE Initiative is available at http://www.strobe-statement.org.
